# Supplementary material for: Feasibility of local anesthesia for full-endoscopic spine surgery: A retrospective cohort study of body weight-based thresholds
Source: PLoS One. 2026 May 29;21(5):e0349672. doi: 10.1371/journal.pone.0349672 (PMC13221022; doi:10.1371/journal.pone.0349672)
Supplement: Attachment — Submitted filename: Response to Reviewers.docx [file pone.0349672.s003.docx]

**Response to Reviewers**

Dear Editor and Reviewers,

We would like to thank you for the thoughtful and constructive feedback on our manuscript entitled "Safety and feasibility of local anesthesia for full-endoscopic spine surgery: A retrospective cohort study of body weight-based thresholds."

We have carefully reviewed each comment and have revised the manuscript accordingly. Specifically, we have adopted a more cautious tone in our conclusions, clarified our local anesthesia safety thresholds relative to FDA labeling, and provided detailed pharmacological and anatomical justifications for our observations. Below are our point-by-point responses to the comments.

**Changes in Authorship**

We would like to inform the Editorial Office that Dr. Atsushi Teramoto has been added as a co-author to this revised manuscript. Dr. Teramoto provided significant intellectual contributions during the revision process, particularly in the critical appraisal of the pharmacological and anatomical interpretations within the Discussion section. He also provided oversight of the data re-analysis and helped refine the manuscript's tone to address the reviewers’ concerns. All authors, including the newly added co-author, have approved this change and the final version of the manuscript.

**Response to Editor’s Comments**

Comment: The conclusions are stated more strongly than the retrospective design can support.

Response: We have revised the manuscript to adopt a more conservative and cautious tone. Throughout the text, we have replaced "achievement" with "observation" and "inherent safety" with "favorable safety profile". We have also added the phrase "within this study cohort" to acknowledge the limitations of our single-center, retrospective data.

**Response to Reviewer #1**

Point 1: The safety thresholds used (7 mg/kg and 5 mg/kg) exceed the FDA-approved limit of 4.5 mg/kg for plain lidocaine.

Response: We appreciate this critical point. We have added a dedicated section in the Discussion to address this. We explicitly state that our thresholds exceed the FDA-approved labeling for plain lidocaine. However, we provide a pharmacological rationale for the tolerance observed in TF-FESS, noting that the dense fibrous structure of the facet joint capsule may act as a barrier that facilitates the sequestration of the anesthetic, leading to more gradual systemic uptake compared to more vascularized tissues.

Point 2: The retrospective identification of LAST and delirium might be under-reported, and conscious sedation could mask early symptoms.

Response: We agree and have addressed this as a major limitation. We have clarified that our results reflect the absence of "clinically significant" LAST episodes. Furthermore, we have added a statement acknowledging that our conscious sedation protocol (pentazocine and hydroxyzine) could have masked early neurological signs of toxicity. We also noted that the absence of standardized tools like the Confusion Assessment Method (CAM) might have led to an under-detection of hypoactive delirium.

**Response to Reviewer #2**

Point 1: Clarify if the thresholds were calculated based on actual, ideal, or adjusted body weight.

Response: We have clarified throughout the manuscript, including the Abstract , Methods , and Conclusion, that all weight-based thresholds were calculated using actual body weight.

Point 2: The frail group is predominantly composed of mildly frail patients.

Response: We have addressed this in the Discussion and Limitations. We specified that 82.1% of our frail group were categorized as mildly frail (mFI-5 = 2). We now emphasize that our results demonstrate high safety even when applying conservative thresholds (5 mg/kg) to this mildly frail population.

Point 3: Table 3 compares heterogeneous populations (different ages and techniques).

Response: To address the statistical uncertainty of our 0% event rate, we have added 95% confidence intervals (0.0–3.4%) to Table 3. We have also added a caveat in the Discussion stating that "direct comparison is limited by differences in patient age and surgical techniques" among the cited studies.

We believe that these extensive revisions, which incorporate more rigorous statistical analysis and a refined pharmacological discussion, have significantly strengthened the scientific rigor and clinical relevance of our study. We hope that the revised manuscript now meets the high standards for publication in *PLOS ONE*. Thank you once again for the opportunity to improve our work.

Sincerely,

Koichi Sairyo, MD, PhD Corresponding Author Department of Orthopedics, University of Tokushima 3-18-15 Kuramoto, Tokushima 770-8503, Japan E-mail: sairyokun@hotmail.com
